# MOF-Integrated Self-Healing Schiff Base Hydrogel for Antibacterial and Antioxidant Wound Treatment

**DOI:** 10.3390/ijms27114726

**Published:** 2026-05-24

**Authors:** Pengyi Zhao, Rui Zhu, Chengxiang Wang, Lei Wang, Hua-Jun Shawn Fan

**Affiliations:** 1 Sichuan University of Science & Engineering, Zigong 643002, China; 18231615631@163.com (P.Z.); zhu-rui0723@icloud.com (R.Z.); 15621137896@163.com (C.W.); 322086001105@stu.suse.edu.cn (L.W.); 2 School of Natural Sciences and Mathematics, Claflin University, 400 Magnolia St, Orangeburg, SC 29115, USA

**Keywords:** self-healing hydrogels, MOFs, curcumin, wound dressings, hyaluronic acid

## Abstract

Chronic wound healing disorders are closely associated with microenvironmental imbalance, while traditional dressings fail to meet dynamic therapeutic demands due to limited functionality, poor responsiveness, and lack of controlled drug release. In this study, a smart hydrogel dressing was developed by integrating curcumin/Cu^2+^ co-loaded UiO-66-NH_2_ metal–organic frameworks into a dynamically cross-linked oxidized hyaluronic acid/carboxymethyl chitosan (OHA-CMCS) network via Schiff base bonding. The MOFs served as a “one-carrier-dual-function” platform, enabling simultaneous delivery of Cu^2+^ and curcumin. The resulting Cur/Cu-MOF@OHA-CMCS hydrogel exhibited a porous structure, excellent self-healing ability, injectability, and favorable rheological and mechanical properties. Additionally, it showed pH-responsive degradation behavior and sustained drug release (~71% within 7 days). The hydrogel demonstrated effective anti-bacterial activity against both *Escherichia coli* and *Staphylococcus aureus*, along with good cytocompatibility (>70% cell viability). These results highlight its potential as a multifunctional and responsive dressing for chronic wound management.

## 1. Introduction

As the largest organ system in the human body and the primary barrier to external aggression, skin tissue plays a key role in maintaining homeostasis of the internal environment. The long-term exposure of the skin to the complex external environment makes it the organ most likely to be traumatized [1]. The diagnosis and treatment of chronic non-healing wounds on the body surface have always been full of challenges, imposing a huge burden on medical care and society [2]. Poor wound healing not only significantly prolongs the treatment period but can also lead to serious infections and even life-threatening conditions. Current clinical treatment strategies include surgical debridement, skin grafting and anti-infective drug interventions [3,4]. Wound dressings play an irreplaceable role in tissue repair through the mechanisms of physical barrier formation, wound exudate management and infection prevention and control. However, with the deepening of the concept of precision medicine, the development of smart dressings with active repair function has become an important research direction in the field of biomaterials. Current wound dressings can be broadly classified into two types: traditional dressings and novel dressings. Traditional dressings (gauze, bandages, cotton balls, etc.) have cost advantages but have inherent defects such as adhesion to the wound, poor permeability and limited protective efficacy [5]. The continuous development of science and technology has led to the emergence of new wound dressings, including film dressings, foam dressings, hydrogel dressings and bioactive dressings [6]. These new wound dressings should not only have the basic physical protection function of traditional wound dressings but also have innovative performance enhancements and incorporate a variety of effects while strengthening the basic functions. The concept of self-healing is derived from the self-healing ability in biology, and most organisms can heal and regenerate themselves after external damage. Inspired by the self-healing phenomena of organisms (e.g., DNA repair, epidermal regeneration, etc.) [7], self-healing hydrogels achieve structural and functional reconstruction of materials after damage by mimicking the damage response mechanism of living systems. Schiff bases can reversibly react under mild conditions, allowing the hydrogel to rapidly recover its structure and function after damage [8]. In addition, the pH-responsive properties of Schiff base hydrogels allow them to undergo network structure dissociation in the mildly acidic environment of bacterial infection, further releasing the active materials loaded into the hydrogel. Therefore, Schiff base hydrogels have excellent drug delivery capabilities and can be loaded with antibiotics [9], metal nanoparticles [10], self-assembled nanoparticles [11,12] and other drugs. Chen et al. [13] combined antibiotics with amino groups and protocatechuic acid with aldehyde groups via Schiff base bonding and verified that the amino groups on the surface of the drug correlated with its loading. This chemically bound loading of the drug in a hydrogel can significantly improve the release behavior of the drug. Hydrogels have been the focus of wound healing dressing research in the last decade, and self-healing polysaccharide hydrogel systems based on dynamic imine bonding have shown unique advantages in the field of wound repair [14]. Hydrogels prepared based on the Schiff base reaction have several advantages such as injectability, self-healing, in situ forming and good biocompatibility, and have been widely used in the fields of drug carriers and cell culture [15,16,17,18,19]. For example, chitosan-oxidized konjac–glucan composite hydrogel was adapted to irregular wounds by injectable molding with good adhesion, self-healing ability and biocompatibility, which significantly shortened the wound healing cycle [20]. In terms of drug delivery, the ethylene glycol chitosan/polyethylene glycol self-healing hydrogel could achieve slow release of paclitaxel with a longer release time than the traditional Pluronic F127 hydrogel system. In addition, the dopamine-modified sodium alginate–polyacrylamide system combines high tensile strength and super ductility through the synergistic action of multiple dynamic bonds (hydrogen bonding and Schiff base bonding), and many catechol groups on the OSA-DA chain can provide the hydrogel with unique cell affinity and tissue adhesion [21]. Such materials have been extended to hemostasis, anti-tumor and cartilage repair [22,23], marking the evolution of self-healing hydrogels towards functional integration.

In response to the dynamic changes in the microenvironment of chronic wounds and the need for stepwise treatment, this study proposes a three-dimensional construction strategy of ‘dynamic network support, intelligent carrier response and active molecule synergy’. Hyaluronic acid (HA) has abundant hydroxyl and carboxyl groups on its main chain, which can be oxidized to form aldehyde groups or modified by acylation to form acyl groups, providing active sites for the Schiff base reaction. However, hyaluronic acid suffers from poor stability, short half-life and susceptibility to degradation, which limits its clinical application [24,25]. By modifying hyaluronic acid prior to the reaction, the above shortcomings can be overcome, allowing hyaluronic acid to have a wider range of biomedical applications. Etherification of many hydroxyl and amino groups within chitosan yields water-soluble carboxymethyl chitosan (CMCS), which has good antimicrobial and anticancer activities, good biocompatibility and superior biodegradability properties. In addition, metal–organic frameworks (MOFs), which are crystalline porous materials formed by coordination of metal nodes and organic ligands, are considered ideal drug carriers due to their excellent antimicrobial properties, good biocompatibility, controlled drug release and high drug loading. Curcumin, a diketone compound with good biocompatibility and biodegradability, is potentially useful in the prevention and treatment of a wide range of diseases due to its diverse effects such as anti-inflammatory [26], antimicrobial [27], antioxidant [28] and anticancer properties [29]. However, the poor water solubility and low oral bioavailability of curcumin have limited its use in clinical practice.

**Figure 1:**
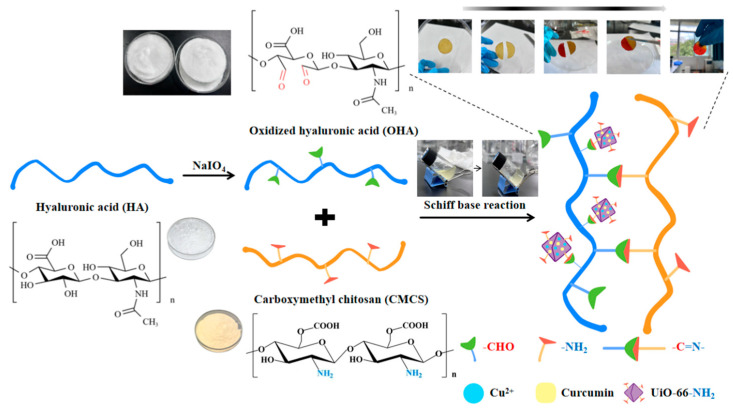
Schematic illustration of the synthesis process of Cur/Cu-MOF@OHA-CMCS composite hydrogel.

As shown in Figure 1, in this study, a novel self-healing hydrogel dressing, Cur/Cu-MOF@OHA-CMCS, was designed to achieve the synchronous co-loading of copper ions (Cu^2+^) and curcumin (Cur), mainly through the construction of amino-functionalized UiO-66-NH_2_ supports: Cu^2+^ accelerates tissue regeneration by regulating the expression of angiogenesis-related proteins; Cur improves the wound microenvironment by inhibiting the release of inflammatory factors with bacterial community sensing and then combines the dynamic Schiff base cross-linked network of oxidized hyaluronic acid (HA) and carboxymethyl chitosan (CMCS) with the bacterial environment and the dynamics of Schiff base bonding to enable the slow on-demand release of Cur/Cu-MOF at the wound site. The morphological structure and chemical composition of the Cur/Cu-MOF material were then investigated, while the micromorphology, rheological properties, self-healing properties, swelling and degradation behavior, and drug release of the hydrogel were studied. The cytotoxicity experiments proved that the hydrogel was non-toxic and harmless and did not affect the normal behavior of cells; the antimicrobial performance of the hydrogel wound dressing drug delivery system was evaluated by in vitro antimicrobial tests to explore its potential as a new medical material.

## 2. Results

### 2.1. Gelation Time

The gelation time of hydrogels prepared with different OSA/CMCS volume ratios (1:1, 1:2, and 2:1) was measured by the vial tilting method. As shown in Table 1, the gelation time decreased by approximately 46 s when the ratio changed from 1:1 to 1:2 and increased by about 21 s when the ratio was adjusted to 2:1. The gelation times of hydrogels with all three volume ratios remained around 5 min, indicating that the hydrogels in all groups can successfully form stable three-dimensional network structures.

### 2.2. Cur/Cu-MOF Morphology and Chemical Composition

**Figure 2:**
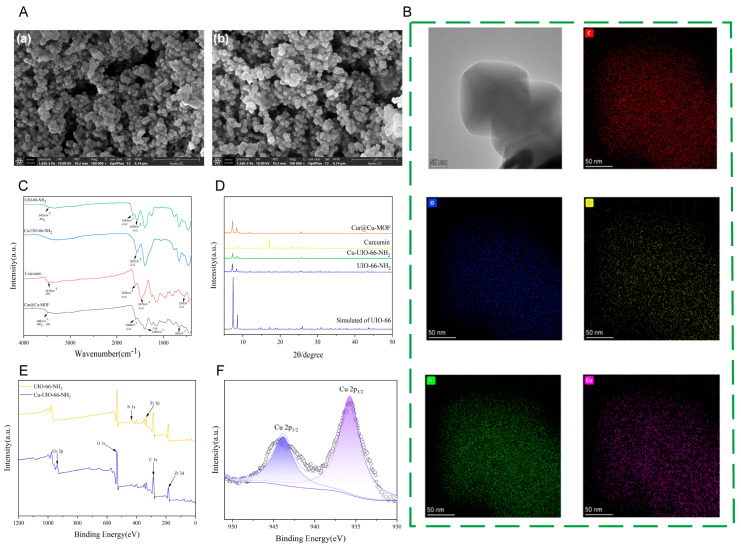
Synthesis and characterization of Cu-UiO-66-NH_2_ and Cur@Cu-UiO-66-NH_2_. (**A**) SEM images of UiO-66-NH_2_ (**a**) and Cu-UiO-66-NH_2_ (**b**); (**B**) TEM and EDS elemental mapping images of Cu-UiO-66-NH_2_; (**C**) FTIR spectra of UiO-66-NH_2_, Cu-UiO-66-NH_2_, curcumin, and Cur/Cu-MOF; (**D**) XRD patterns of UiO-66-NH_2_, Cu-UiO-66-NH_2_, curcumin, and Cur/Cu-MOF; (**E**) XPS spectra of UiO-66-NH_2_ and Cu-UiO-66-NH_2_; and (**F**) Cu 2p XPS spectra of Cu-UiO-66-NH_2_.

UiO-66-NH_2_ was successfully prepared by the solvothermal method using 2-aminoterephthalic acid and zirconium tetrachloride as precursors and subsequently impregnated in copper nitrate solution to obtain Cu-UiO-66-NH_2_ by post-synthetic modification strategy. As shown in Figure 2A, the SEM characterization showed that the prepared UiO-66-NH_2_ exhibited a regular polyhedral morphology with a smooth surface and well-defined angles. SEM imaging revealed the presence of agglomeration in the particles, which may be due to van der Waals force interactions induced by high-energy states on the MOF surface.

As shown in Figure 2B, the copper-doped Cu-UiO-66-NH_2_ still retains complete polyhedral geometry without any obvious etching or morphological collapse, indicating that the post-modification process has not damaged the crystal framework of the MOF. In addition, the uniform spatial distribution of Cu elements in the MOF supports can be clearly observed by surface scanning EDS analysis.

Infrared spectroscopy analysis provided an important basis for revealing the structural properties and interaction mechanisms of the materials. As shown in Figure 2C, the characteristic peaks of UiO-66-NH_2_ at 3455 cm^−1^, 1643 cm^−1^ and 1566 cm^−1^ are consistent with the typical features of aminated metal–organic frameworks. The Cu-UiO-66-NH_2_ formed by the introduction of copper ions retains the original aromatic ring vibrational features at 1567 cm^−1^, indicating that the metal-node modification has not destroyed the basic backbone structure of the ligand. Notably, when curcumin was loaded on Cu-UiO-66-NH_2_ to form the Cur/Cu-MOF complex, the N-H vibrational peak was shifted to 3482 cm^−1^, while the C=O vibrational peak was shifted to 1580 cm^−1^ and a new carboxylate symmetric telescoping vibrational peak appeared at 1405 cm^−1^. These changes indicate the existence of hydrogen bonding between the amino group and the curcumin molecule, while the π-π stacking effect between its conjugated system and the MOF aryl ring results in the redshift of the characteristic peak. In addition, the emerging peak at 662 cm^−1^ can be attributed to the vibration of the Cu-O bond, further confirming the coordination bond between the metal node and the curcumin molecule.

The crystal structure of the material was systematically characterized by XRD analysis. As shown in Figure 2D, the sharp diffraction peaks of UiO-66-NH_2_ at 7.19°, 8.27° and 25.74°, whose peak positions essentially coincide with the standard analog spectrum of UiO-66 without peak broadening, confirm that the material has good crystalline integrity. The Cu-UiO-66-NH_2_ formed by Cu doping did not show any significant shift in the main diffraction peaks, indicating that the introduction of a low concentration of Cu ions did not disrupt the framework integrity of the MOF. It is worth noting that in the Cu-doped Cu-UiO-66-NH_2_ sample, the diffraction peaks of (111) and (002) crystal planes located in the low-angle interval of 5–10° are shifted to the low-angle direction, and this change may be due to the local bond length adjustment caused by the coordination interaction between copper ions and zirconium–oxygen clusters, which in turn triggers the lattice aberration, although the overall framework structure is still intact.

The chemical states of the metal elements in the material were analyzed in detail by XPS. As shown in Figure 2E,F, the Cu 2p fine spectrum of Cu-UiO-66-NH_2_ exhibits typical double peaks at 935.78 eV and 943.89 eV, which are attributed to the characteristic peaks of the 2p_3/2_ and 2p_1/2_ orbitals of Cu^2+^, respectively. It is noteworthy that the binding energies of the Zr 3d, C 1s and N 1s characteristic peaks of the Cu-modified material are not significantly shifted compared to those of UiO-66-NH_2_, suggesting that the introduction of Cu neither changes the coordination environment of the zirconium–oxygen clusters nor significantly affects the distribution of the carbon and nitrogen elements of the organic ligands in terms of their electron cloud. The above results indicate that the successful construction of Cur/Cu-MOFs is not only based on physical adsorption but also involves multiple synergistic effects such as hydrogen bonding, π-π interactions and metal coordination, which provides a structural basis for the subsequent study of the stability and controlled-release behavior of the drug-loaded system.

### 2.3. OHA Characterization Results

**Figure 3:**
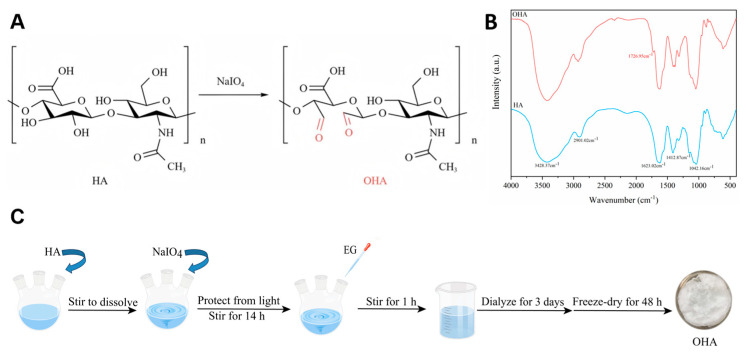
(**A**) Synthesis route of OHA, (**B**) FTIR spectra of HA and OHA and (**C**) the preparation process of OHA.

The mechanism of HA being oxidized by sodium periodate into OHA is shown in Figure 3A. During the oxidation reaction, the adjacent diol structure in the HA molecular chain undergoes selective C2–C3 bond cleavage under the strong oxidation effect of NaIO_4_, and then the cyclic structure is opened and transformed into two aldehyde functional groups (-CHO). The reaction mechanism of the oxidation of HA by NaIO_4_ and the preparation process of OSA are shown in Figure 3B,C. The C-C bond in the o-diol in the structural unit of HA can be oxidized by NaIO_4_ and then cleaved to form an aldehyde group. The FTIR spectra of OHA and HA are used to monitor and illustrate the bond structural changes, as shown in Figure 3C. The absorption peaks at about 3428 cm^−1^ are the stretching vibration peaks of -OH in OHA and HA. HA contains many hydroxyl groups in its structure, which can easily form hydrogen bonds with electron-donating groups such as -OH and -NH_2_, and the more hydrogen bonds there are, the broader the stretching vibration peaks of -OH are. The characteristic absorption peak of HA is 1623 cm^−1^, corresponding to the amid vibration band. The peak at around 1412 cm^−1^ is attributed to the antisymmetric and symmetric telescopic vibrations due to the conjugation of the carboxylate ion (COO-). After oxidation, a new absorption peak was detected at around 1730 cm^−1^ for OHA, which can be attributed to the C=O bending vibration absorption peak in the ketone or aldehyde structure, indicating the partial conversion of the two adjacent hydroxyls on the six-membered ring to aldehyde groups. The FTIR spectra of OHA and HA were very close to each other, probably due to the hemiacetal formation, so that the signal of the aldehyde group was weakly detected. The above results indicate that OHA has been successfully prepared.

### 2.4. Morphological and Infrared Spectroscopic Results and Rheological Properties of Cur/Cu-MOF@OHA-CMCS Hydrogels

**Figure 4:**
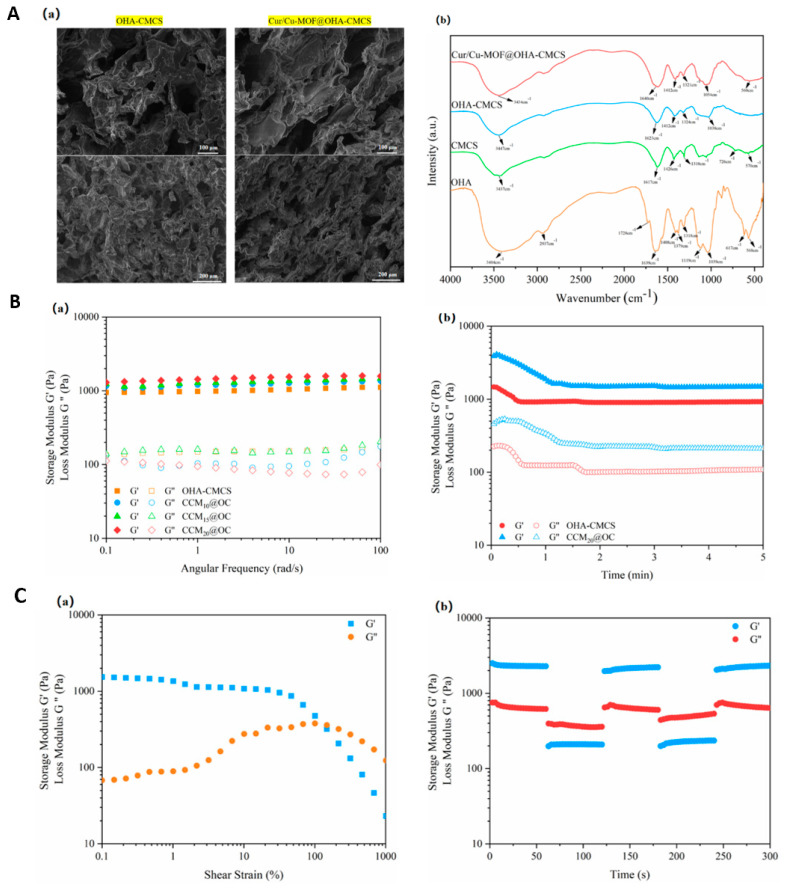
(**A**) SEM images of OSA-CMCS and Cur/Cu-MOF@OSA-CMCS (**a**) and IR spectra of CCM@OC hydrogels (**b**); (**B**) dynamic frequency sweep measurements (**a**) and time sweep measurements of OHA-CMCS and CCM@OC hydrogels (**b**); (**C**) strain amplitude sweep measurements (**a**) and step-strain measurements of CCM20@OC hydrogels (**b**).

The porous structure of the hydrogel 3D network plays an important role in the practical application of the hydrogel as a channel for water vapor and other nutrients to be transported within the hydrogel. As shown in Figure 4(Aa), the hydrogel has a uniform porous network structure, and this hollow porous structure enables the hydrogel to have a good water retention ability, and at the same time makes the hydrogel adhere well to the wound, absorb wound exudate, prevent wound infection, and provide a moist environment for wound healing. OHA-CMCS hydrogel shows a loose porous structure, and after the addition of Cur/Cu-MOF, the hydrogel showed a denser network with reduced pore size.

The results of infrared spectroscopy analysis confirmed the successful construction of the hydrogel network structure by chemical cross-linking between OHA and CMCS. As shown in Figure 4(Ab), there was a distinct aldehyde C=O stretching vibration peak at 1728 cm^−1^ in the characteristic spectrum of OHA, indicating the successful aldolization of hyaluronic acid, whereas the characteristic peak at 1617 cm^−1^ in the CMCS spectrum is attributed to the N-H bending vibration of the primary amine group. When OHA was compounded with CMCS to form a hydrogel, the characteristic peaks of the aldehyde group of OHA (1728 cm^−1^) and the amino group of CMCS (1617 cm^−1^) were significantly weakened, and at the same time a new broad peak appeared at 1623 cm^−1^, which was attributed to the C=N bond stretching vibration generated by the Schiff base reaction. In the composite hydrogel loaded with Cur/Cu-MOF, the characteristic peak at 1640 cm^−1^ is further shifted to a higher wavenumber, which may be related to the coordination interaction of Cu^2+^ with carboxylic acid groups, while the newly appeared weaker peak at 560 cm^−1^ may originate from the metal–organic framework (MOF) vibrations of the Cu-O bond in the MOF. Together, the above spectral variation rules support the hydrogel formation mechanism of three-dimensional network structure through dynamic imine bonding.

The viscoelastic variation curves of the storage modulus G′ and loss modulus G″ of hydrogels with angular frequency are shown in Figure 4B. The angular frequency varies from 0.1 to 100 rad/s. It can be seen that the storage modulus G′ of all hydrogel samples was consistently higher than the loss modulus G″ over the entire scanning frequency range, indicating that the hydrogels exhibited dominant elastic behavior and a stable cross-linked network structure. The storage moduli G′ and G″ of all hydrogel samples are greater than their respective loss moduli G″, demonstrating that the elastic deformation of the hydrogels is greater than their viscous deformation, indicating that the hydrogels have a stable cross-linked structure. The highest values of G′ and G′ were found for CCM_20_@OC, indicating that the introduction of Cur/Cu-MOF had a positive effect on the mechanical properties of the hydrogel, as shown in Figure 4(Ba). The time scans of OHA-CMCS and CCM_20_@OC hydrogels were performed by a rheometer, as shown in Figure 4(Bb). The G′ value of CCM_20_@OC hydrogel finally stabilized at 1482.9 Pa, while the G′ value of OHA-CMCS hydrogel finally stabilized at 922.11 Pa.

The rheological recovery test can investigate the self-healing properties of hydrogels from a microscopic point of view. As shown in Figure 4(Ca), in the range of shear strain from 0.1% to 100%, the changes in storage modulus G′ and loss modulus G″ are not obvious, and the storage modulus G′ is larger than the loss modulus G″, indicating that the hydrogel can withstand the maximum strain. The storage modulus G′ is larger than the loss modulus G″, indicating that the elastic deformation of the hydrogel dominates and the hydrogel structure is relatively stable at this time. As the strain value increases, the storage modulus and loss modulus both decrease significantly. As can be seen from the figure, the two curves cross near γ = 150%; at this time, the storage modulus G′ is equal to the loss modulus G″. As the strain continues to increase, the loss modulus G″ becomes larger than the energy storage modulus G′, indicating that the polymer molecular chain within the hydrogel sample has been significantly altered and the hydrogel cross-linking network has been destroyed. Therefore, γ = 150% can be defined as the strain value corresponding to the gel–sol transition point of the CCM_20_@OC hydrogel sample. The self-healing ability of the CCM_20_@OC hydrogels was then tested by performing dynamic rheological experiments. By determining the sol–gel point, it is possible to take γ = 1% as a small strain and γ = 300% as a large strain, where the hydrogel is destroyed at the large strain and restored at the small strain.

As shown in Figure 4(Cb), at the initial stage, G′ > G″, indicating that the hydrogel can maintain the stability of its own cross-linked network at this time; subsequently, after the shear strain is increased to 300%, G′ < G″, and the energy storage modulus rapidly decreases from 2498.7 Pa to about 396.38 Pa. At this time, the hydrogel network is destroyed by the external force and it shows the viscoelastic state; however, when the large stress of the hydrogel is removed, the energy storage modulus of CCM_20_@OC hydrogel can rapidly recover the mechanical modulus before damage under the condition of small strain γ = 1%, and G′ > G ″ again, indicating that the hydrogel can maintain the stability of its own cross-linked network at this time. Then G″ > G′ again, i.e., the hydrogel returns to the initial stable cross-linked network. This cyclic process verifies the self-healing behavior of the hydrogel.

### 2.5. Swelling, Degradation, Self-Healing, Injectability and Adhesion Behavior of Cur/Cu-MOF@OHA-CMCS Hydrogels

**Figure 5:**
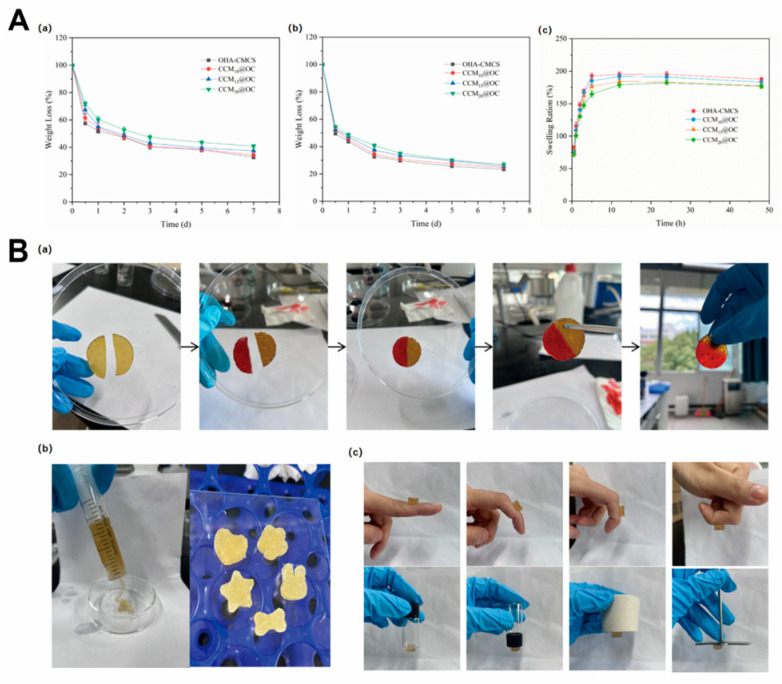
(**A**) Degradation curves of OHA-CMCS and CCM@OC hydrogels in vitro at 37 °C with different pH buffers, pH = 7.4 (**a**) and pH = 5.5 (**b**), and swelling ratio of OHA-CMCS and CCM@OC hydrogels at 37 °C (**c**); (**B**) the self-healing (**a**), injectable (**b**) and adhesion (**c**) behavior of CCM@OC hydrogels.

Degradability is an important metric in the evaluation of hydrogels for tissue repair and wound dressings. Novel wound dressings require materials with good degradability, so we tested the degradability of hydrogel samples in PBS phosphate buffer at pH 7.4. As shown in Figure 5(Aa), the OHA-CMCS hydrogel degraded at the fastest rate and the CCM_20_@OC hydrogel sample degraded at the slowest rate. There are two main factors that influence the degradation behavior of hydrogels: the cross-linking density of the hydrogel and its cross-linking mechanism. The lower the cross-link density, the more pronounced the degradation behavior; also, the combination of chemical cross-linking and physical cross-linking accelerates the hydrogel degradation process. We then tested the degradation ability of the hydrogels in a mildly acidic environment using acetate buffer (pH = 5.5) to simulate the micro-acidic environment at the site of wound inflammation. As shown in Figure 5(Ab), the degradation rate of the hydrogel in the acetate environment at pH 5.5 was significantly higher than that in the PBS phosphate buffer environment at pH 7.4, with a degradation rate of over 60% after 12 h. This shows that the hydrogel has good degradability and can respond to the micro-acidic environment of the wound site, meeting the requirements of new wound dressings.

Hydrogels have a certain ability to absorb and swell in an aqueous environment due to their specific three-dimensional network structure. A good swelling performance can regulate the exchange of substances inside and outside the hydrogel and the ability of drug release. As shown in Figure 5(Ac), the swelling rates of OHA-CMCS, CCM_10_@OC, CCM_15_@OC and CCM_20_@OC hydrogel samples were 195%, 191%, 184% and 179%, respectively, at 12 h with the extension of time. Thereafter, it gradually decreased and finally stabilized. In the early stage, the water absorption capacity plays a dominant role in the swelling rate. In addition, the swelling rate decreased slightly with the increase in Cur/Cu-MOF concentration. In general, swelling behavior is closely related to the cross-link density and hydrophilicity of the material. Therefore, it can be reasonably concluded that the hydrogel network formed in the presence of Cur@Cu-MOF is denser and the MOF has a lower hydration capacity, resulting in a lower water absorption capacity. Nevertheless, the swelling properties of the CCM_20_@OC hydrogel were still sufficient to support the absorption of wound tissue exudate.

To evaluate the self-healing ability of the CCM_20_@OC hydrogel, a macroscopic self-healing test was first performed. As shown in Figure 5(Ba), it can be seen that the cut portion in the middle of the two spliced hydrogels was bonded together after 5 min of rest. At the same time, the interface of the two different-colored hydrogels started to blur, and the dye molecules diffused at the repaired interface and showed mutual penetration, which reflected the re-healing of the interface at the molecular level. Due to the dynamic reversibility of Schiff bases, the self-healing hydrogels prepared by cross-linkers containing aldehyde groups showed good injectability. When the gel was injected from the syringe, the gel would be destroyed by the external shear force, and the gel without self-healing ability might undergo gel–sol transition due to the shear force, whereas the CCM_20_@OC hydrogel could self-heal by regenerating Schiff bases, so that the gel injected into the Petri dish showed an elongated column extruded by the syringe. As shown in Figure 5(Bb), the excellent injectable properties of the hydrogel were macroscopically demonstrated. In addition, the custom silicone mold method was used to achieve controlled molding of the complex shape of the hydrogel.

As shown in Figure 5(Bc), the CCM_20_@OC hydrogel has excellent adhesion properties and can adhere to a variety of materials with different substrate shapes, including non-biological surfaces (glass, plastic, rubber, stainless steel, etc.) and biological tissues (skin, etc.). In conclusion, hydrogels with shape conformability, self-healing and injectability can guide tissue regeneration through minimally invasive implantation and perfectly fill irregular wounds to accelerate their healing.

### 2.6. Drug Release Properties of Cur/Cu-MOF@OHA-CMCS Hydrogel

**Figure 6:**
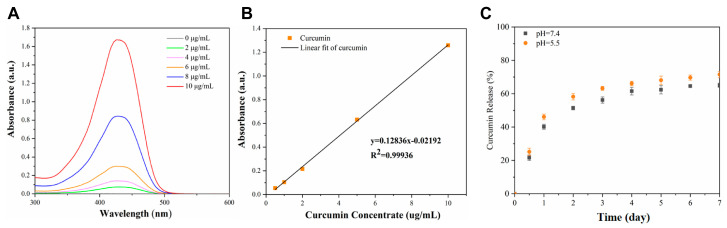
(**A**) UV–Vis spectra of curcumin at different concentrations; (**B**) relationship between absorption intensity and curcumin concentration; (**C**) cumulative release of curcumin from CCM20@OC hydrogel.

The absorbance of curcumin standard solution at 425 nm at different concentrations was measured by a UV spectrophotometer (Figure 6A), and the standard absorption curve of curcumin concentration (Figure 6B) was plotted and fitted as a function of absorbance (*Y*-axis) and concentration (*X*-axis). The equation of the standard curve was obtained as follows:

y = 0.12836x − 0.02192, R^2^ = 0.99936.

This shows that there is a good linear relationship between absorbance intensity and curcumin concentration.

As shown in Figure 6C, the cumulative release percentage of curcumin was calculated from the standard curve of curcumin to plot the release curve of CCM_20_@OC hydrogel in PBS buffer of different pH. The cumulative release percentage of curcumin increased rapidly from 0 to 48 h. The cumulative release percentages were 51.39% and 58.21% at 48 h, respectively. After that, it increased slowly from 48 to 168 h and then leveled off to reach a maximum cumulative release percentage of 65.07% and 71.52%, respectively, which can be considered as a sustained drug release. The rate of Cur release from CCM_20_@OC hydrogel in acetate buffer (pH = 5.5) medium was higher than that in PBS phosphate buffer (pH = 7.4) due to the fact that CCM_20_@OC hydrogel is able to trigger the dissociation of Schiff base bonds under slightly acidic (pH = 5.5) conditions, which degrades the hydrogel and allows the release of internally loaded antimicrobial drugs, and due to its excellent pH responsiveness to respond to the slightly acidic environment of bacterial infections.

### 2.7. Cytotoxicity Test Results and Antibacterial Properties of Cur/Cu-MOF@OHA-CMCS Hydrogel

**Figure 7:**
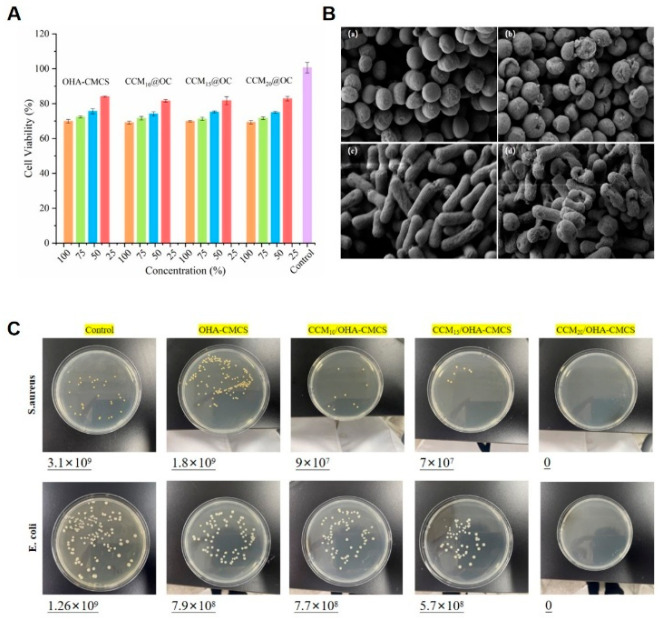
(**A**) Cytocompatibility results of OHA-CMCS and CCM@OC hydrogels; (**B**) SEM images of *S. aureus* incubated (**a**) without and (**b**) with CCM_20_@OC hydrogel-treated medium and SEM images *E. coli* incubated (**c**) without and (**d**) with CCM_20_@OC hydrogel-treated medium; and (**C**) images of bacterial colonies after co-culture of CCM@OC hydrogels.

The CCK-8 method was used to test the cytocompatibility of the hydrogel extracts, and the results of the cell survival rate of different concentrations of OHA-CMCS, CCM_10_@OC, CCM_15_@OC and CCM_20_@OC hydrogel extracts treated for 48 h are shown in Figure 7A. The survival rate of BEAS-2B cells reached more than 70% in the different concentration hydrogel treatment groups compared to the control group.

Bacterial infection is the most common and almost inevitable problem in the wound healing process. When a wound becomes infected, bacteria can cause a prolonged inflammatory response at the site of infection, further delaying the healing process during the inflammatory phase. To characterize the antimicrobial properties of the hydrogel dressing system, *Staphylococcus aureus* (*S. aureus*) and *Escherichia coli* (*E. coli*) were selected as test bacteria. Plate colony counting experiments (Figure 7C) showed that OHA-CMCS, CCM_10_@OC, CCM_15_@OC and CCM_20_@OC hydrogels co-cultured with the bacterial solution for 24 h produced *E. coli* and *S. aureus* colonies with counts of 1.8 × 10^9^, 9 × 10^7^, 7 × 10^7^, and 0, and 7.9 × 10^8^, 7.7 × 10^8^, 5.7 × 10^8^, and 0, respectively, and CCM_20_@OC hydrogel had more significant antimicrobial properties against *E. coli* and *S. aureus*. *Staphylococcus aureus* and CCM^20^@OC hydrogel have more significant antibacterial properties. The morphological changes of the bacteria before and after treatment with CCM_20_@OC hydrogel were further analyzed, as shown in Figure 7B. In the control group without the addition of CCM_20_@OC hydrogel, *E. coli* and *S. aureus* had intact cell morphology and no change in cell morphology was observed. However, *S. aureus* treated with CCM_20_@OC hydrogel showed crumpling of bacterial cells and rupture of cell membranes; CCM_20_@OC hydrogel treated *E. coli*. This was mainly due to the fact that the CCM_20_@OC hydrogel responded to the stimulation of the CCM_20_@OC hydrogel via the dual pH/temperature release mechanism, in which the amino group of the MOF dissociates from CMCS via dynamic imine bonding in a mildly acidic infection microenvironment (pH = 5.5–6.5), triggering the targeted release of Cu^2+^ and Cur. This multi-layered synergistic mechanism enables this hydrogel to demonstrate efficient and long-lasting antimicrobial potential in the treatment of complex infected wounds.

## 3. Discussion

### 3.1. Analysis of Gelation Time

In this work, hyaluronic acid (HA) was selectively oxidized with sodium periodate (NaIO_4_) as a strong oxidant, whereby partial vicinal hydroxyl groups on the six-membered ring of HA were oxidized to aldehyde groups. The obtained dialdehyde structure of oxidized hyaluronic acid (OHA) imparts enhanced cross-linking capacity, allowing OHA to simultaneously interact with multiple amino groups of CMCS molecules. With the increase in CMCS proportion, the concentration of free amino groups in the system elevates, which raises the collision probability between amino and aldehyde groups and thus remarkably accelerates the formation of cross-linked networks, corresponding to a shortened gelation time. In contrast, a higher OHA proportion increases the total amount of aldehyde groups; nevertheless, the relatively insufficient amino groups supplied by CMCS restrict the intermolecular cross-linking efficiency, thereby delaying the gelation process. Although the gelation time varies with the volume ratio of the two components, all hydrogel groups exhibit a gelation time of approximately 5 min, demonstrating that stable three-dimensional network structures can be successfully constructed for all formulations.

### 3.2. Structural Evolution of MOF Materials

The observed particle agglomeration can be attributed to the high surface energy of MOF nanoparticles, which induces strong van der Waals interactions between particles. The well-preserved morphology after Cu modification indicated that post-synthetic metal doping did not destroy the crystalline skeleton, which is crucial for maintaining structural stability and pore structure. Uniform Cu distribution suggested effective coordination between Cu ions and the Zr–O clusters or ligands.

FTIR results verified the intact framework of UiO-66-NH_2_ after Cu introduction. Peak shifts after curcumin loading suggested the formation of hydrogen bonds between –NH_2_ of the MOF and curcumin. The π–π stacking between the conjugated structure of curcumin and aromatic rings of the ligand also contributed to the peak shifts. The emerging Cu–O peak confirmed the coordination interaction between Cu nodes and curcumin, indicating that drug loading involved multiple interactions rather than simple physical adsorption.

XRD patterns confirmed the high crystallinity of UiO-66-NH_2_. The unchanged main peaks after Cu doping indicated that low-concentration metal incorporation did not disrupt the long-range framework. The slight low-angle shift was caused by local bond length adjustment and slight lattice distortion due to Cu–Zr–O coordination, while the overall structure remained stable.

XPS results confirmed the presence of Cu^2+^ and showed negligible changes in Zr, C, and N chemical states, demonstrating that Cu modification did not alter the coordination environment of Zr–O clusters or the electron distribution of organic ligands. The above results collectively proved that the construction of Cur/Cu-MOF involved hydrogen bonding, π–π stacking, and metal–ligand coordination, providing a stable basis for controlled drug release.

### 3.3. Structural Characteristics of Oxidized Hyaluronic Acid

The appearance of the aldehyde C=O peak at 1730 cm^−1^ proved the successful oxidation of HA to OHA via selective cleavage of the o-diol structure under NaIO_4_ oxidation. The similar overall FTIR profiles between HA and OHA were due to hemiacetal formation, which weakened the aldehyde signal. The introduction of aldehyde groups enabled the subsequent Schiff base reaction with amino groups, laying the foundation for the formation of dynamic cross-linked hydrogels.

### 3.4. Network Formation and Viscoelastic Properties of Hydrogels

The denser pore structure after MOF incorporation indicated that Cur/Cu-MOF acted as a hybrid cross-linking node, reinforcing the hydrogel network. The enhanced mechanical strength (higher G′) can be attributed to the synergistic effect of Schiff base cross-linking between OHA and CMCS and additional interactions between MOF particles and polymer chains, such as hydrogen bonding and electrostatic attraction. The self-healing behavior was enabled by the dynamic reversibility of the Schiff base bonds, allowing the hydrogel to recover its network structure after damage.

### 3.5. Swelling, Degradation, and Functional Properties

The pH-dependent degradation behavior suggested that the hydrogel could respond to the acidic microenvironment of infection sites, which is beneficial for on-demand drug release. The reduced swelling ratio with increasing MOF content was due to the enhanced cross-linking density, which limited the water uptake capacity of the network. The excellent injectability and adhesion further confirmed its suitability for in vivo applications.

### 3.6. pH-Responsive Drug Release

The faster release in acidic medium was mainly caused by the protonation of the MOF surface and the weakening of hydrogen bonding and coordination interactions between curcumin and the MOF, promoting drug diffusion. The sustained release behavior in neutral medium could reduce systemic side effects and prolong the therapeutic time window.

### 3.7. Biological Performance

The high cell viability indicated that the hydrogel had good cytocompatibility, meeting the basic requirement for biomedical materials. The significant antibacterial activity of CCM20@OC was attributed to the combined effect of Cu^2+^ release and curcumin, which disrupted bacterial cell membranes and induced oxidative stress, leading to bacterial death.

## 4. Materials and Methods

### 4.1. Materials

2-aminoterephthalic acid (H_2_BDC), zirconium tetrachloride (ZrCl_4_), N, N-Dimethylformamide (DMF), curcumin (Cur), a dialysis bag (Mw = 3500), hyaluronic acid (HA, 5–150 kDa) and carboxymethyl chitosan (CMCS, 250–400 kDa) were purchased from Shanghai Titan Scientific Co., Ltd., Shanghai, China. Ethanol (EtOH), methanol (MeOH), ethylene glycol (EG) and sodium chloride (NaCl) were purchased from Cheng-du Kelong Chemical Co., Ltd., Chengdu, China. BEAS-2B, *Escherichia coli* (*E. coli*) and *Staphylococcus aureus* (*S. aureus*) were purchased from China Center of Industrial Culture Collection, Beijing, China. Cu (NO_3_)_2_·3H_2_O was purchased from Xi-long Scientific Co., Ltd., Shantou, China. Deionized water was prepared by our laboratory. Phosphate buffered saline (PBS, pH = 7.4) and sodium acetate–acetic acid buffer solution (Hac-NaAc, pH = 5.5) were purchased from Phygene Biotechnology Co., Ltd., Fuzhou, China. Sodium periodate (NaIO_4_), tryptone, yeast extract powder and agar were purchased from Aladdin Biochemical Technology Co., Ltd., Shanghai, China. Culture medium and Cell Counting Kit-8 (CCK-8) were purchased from Beyotime Biotechnology, Shanghai, China.

### 4.2. Preparation of Metal–Organic Frame Material UiO-66-NH_2_ and Cur/Cu-MOF

First, 347.4 mg of zirconium tetrachloride (ZrCl_4_) was dissolved in 30 mL of dimethylformamide (DMF) and ultrasonicated for 20 min to obtain a clear solution at room temperature. Then, 246.3 mg of terephthalic acid (H_2_BDC) was added to the solution and ultrasonicated for another 20 min. The mixed solution was then transferred into a 50 mL Teflon-lined stainless-steel autoclave, sealed, and heated in an oven at 120 °C for 24 h. After the reaction, the mixture was centrifuged at 10,000 r/min for 10 min to collect the crude solid. The solid was washed with DMF and methanol (MeOH) three times, and after each washing step, the product was harvested by centrifugation at 10,000 r/min for 10 min to remove unreacted ligands and metal ions. Finally, the product was dried under vacuum at 80 °C for 12 h to obtain UiO-66-NH_2_ nanoparticles. The as-prepared sample was stored in a glove box for subsequent experiments.

Next, at room temperature, 608 mg of Cu (NO_3_)_2_-3H_2_O was dissolved in 50 mL of EtOH, then 200 mg of the prepared MOF (UiO-66-NH_2_) was added, and the resulting mixture was stirred for 3 h. The solids were collected by centrifugation to obtain the Cu-MOF. During the curcumin encapsulation process, Cu-UIO-66-NH_2_ was added into curcumin ethanol solution (1 g/L) in a proper solid–liquid ratio for adsorption. The Cu-MOF was then added to the ethanol solution of curcumin (Cur) (50 mg/mL), and the mixture was left in the dark for 12 h to fully load the curcumin molecules into the pores of the Cu-MOF. The corresponding centrifugation condition was 10,000 r/min for 10 min. The prepared Cu-MOF and Cur/Cu-MOF samples were stored in a dry environment for subsequent characterization and application studies.

### 4.3. Preparation of Oxidized Hyaluronic Acid (OHA)

First, 5 g of hyaluronic acid (HA) was dissolved in 200 mL of deionized water, and after complete dissolution, 3.5 g of sodium periodate (NaIO_4_) was added and the reaction was stirred for 14 h at 25 °C and protected from light, followed by the addition of 5 mL of ethylene glycol (EG) to complete the reaction, and stirring was continued for 1 h. At the end of the reaction, the solution was dialyzed using a dialysis bag with a molecular weight cut-off of 3500 (Mw = 3500) in deionized water for 3 d to purify the product, during which time the dialysis water was changed at least three times a day. Finally, the product OHA was obtained by lyophilization for 48 h.

### 4.4. Preparation of Cur/Cu-MOF@OHA-CMCS Hydrogels

OHA-CMCS hydrogels were prepared by mixing 10% (*w*/*v*) OHA solution and 5% (*w*/*v*) CMCS solution in a 1:2 (*v*/*v*) ratio at room temperature. Subsequently, Cur/Cu-MOF@OHA-CMCS hydrogels were prepared by homogeneously doping 10, 15 and 20 mg Cur/Cu-MOF into the CMCS-OHA hydrogels, designated CCM_10_@OC, CCM_15_@OC and CCM_20_@OC, respectively. Notably, the hydrogels were not subjected to post-gelation purification or drying unless otherwise specified.

### 4.5. Characterization

#### 4.5.1. Gelation Time

The gelation time was determined by a simple vial-inversion method. At room temperature, a carboxymethyl chitosan solution was poured into a glass vial, followed by the addition of varying volumes of an oxidized hyaluronic acid solution, and the timer was started immediately. The vial was gently inverted at regular intervals to monitor the flow behavior of the mixture. As the reaction proceeded, the solution gradually lost its fluidity and transformed into a non-flowing gel; the elapsed time at which the meniscus no longer moved upon inversion was recorded as the gelation time, providing a qualitative measure of the gelation rate and rapid-setting capability of the hydrogel.

#### 4.5.2. Fourier Transform Infrared Spectroscopy (FTIR)

The chemical functional groups of the samples were analyzed by a Bruker ALPHA II FTIR spectrometer (Bruker, Karlsruhe, Germany). All spectra were recorded for 32 scans in the transmission mode in the range of 4000–400 cm^−1^ with a resolution of 4 cm^−1^.

#### 4.5.3. Morphology (SEM)

The cross-sectional morphology of the hydrogel was observed using a SEM 5000 field-emission scanning electron microscope (CIQTEK, Hefei, China) at an acceleration voltage of 2 kV. SEM characterization of the as-prepared MOF was performed at an accelerating voltage of 10 kV (Thermo Fisher Scientific Apreo 2C, OXFORD ULTIM Max65, Waltham, MA, USA). The micrographs were captured at magnifications of 100,000× to observe the overall morphology and fine microstructure of MOF particles.

#### 4.5.4. X-Ray Diffraction (XRD)

The crystal structure of UiO-66-NH_2_, Cu-UiO-66-NH_2_, Curcumin, and Cur/Cu-MOF was determined with a Rigaku D/Max-1200 (Rigaku, Tokyo, Japan) in the 2θ angle range of 0–70.0°.

#### 4.5.5. X-Ray Photoelectron Spectrometer (XPS)

The surface Cu atomic percentage was determined by XPS (Thermo Fisher Scientific ESCALAB 250Xi, Waltham, MA, USA).

#### 4.5.6. Transmission Electron Microscopy (TEM)

The morphology of Cu-UiO-66-NH_2_ in the hydrogel was imaged with a JEM 2100F transmission electron microscope (JEOL, Tokyo, Japan) at the working voltage of 200 kV.

#### 4.5.7. Rheological Measurements and Self-Healing Performance

Rheological behavior of the hydrogels was probed on an Anton Paar Physica MCR 301 rheometer (Artisan Technology Group, Graz, Austria) by performing an oscillatory frequency sweep at 25 °C, maintaining a constant strain amplitude of 1% over the angular frequency window 0.1–100 rad s^−1^.

The ability of self-healing performance was quantified by a step-strain sweep: the sample was alternately subjected to a small strain (γ = 1%, 1 Hz) for structural recovery and a large strain (γ = 300%, 1 Hz) for rupture. Each segment lasted at 3 s with 20 data points, giving a total of 300 s. The evolution of G′ and G″ across cycles was used to assess self-healing capability.

#### 4.5.8. Swelling Properties

The freezing-dried hydrogels with diameters of 10 mm and thicknesses of 2 mm were immersed in phosphate-buffered saline (PBS, pH 7.4), allowed to swell, and weighed after the excessive surface water was removed. The swelling ratio was calculated using Equation (1).

(1) $$Q=\frac{m_{t}-m_{0}}{m_{0}},$$

where m_t_ is the mass of hydrogel at the swelling time t, and m_0_ is the initial mass of hydrogel.

#### 4.5.9. Degradation Properties

The freezing-dried hydrogels with diameters of 10 mm and thicknesses of 2 mm were individually immersed in pH 7.4 PBS (normal physiological condition) and pH 5.5 acetate buffer (inflammatory acidic condition) and incubated at 37 °C for 7 days to assess in vitro degradation. At predetermined time points (0, 0.5, 1, 2, 3, 5, 6, and 7 days), samples were withdrawn, lyophilized to constant weight, and accurately weighed with an analytical balance (n = 3). The percentage mass loss was calculated using Equation (2).

(2) $$D=\frac{\left(w_{0}-w_{t}\right)}{w_{0}},$$

where w_0_ is the initial dry weight and w_t_ is the dry weight at time t.

#### 4.5.10. Self-Healing, Injectability, and Adhesion of the Hydrogel

CCM_20_@OC hydrogel was first bisected with a razor blade; one half was immersed in red dye for tracking (dyeing does not affect hydrogel performance). To mimic damage, the two halves were brought back into contact without any external pressure, and macroscopic self-healing was observed. Next, the hydrogel was loaded into a syringe and extruded through a needle into a Petri dish; the extruded gel rapidly re-integrated into a single piece. Additionally, the gel was injected into a silicone mold cavity, demolded after a short rest, and retrieved as a well-defined shape. Meanwhile, pre-formed CCM_20_@OC samples were pressed onto various substrate surfaces to assess their adhesive behavior.

#### 4.5.11. Drug Release Properties

The curcumin release profile of the CCM@OC hydrogel was assessed using a dialysis-bag replacement method. A calibration curve of curcumin was first constructed in 50% ethanol-containing corresponding buffer (1–10 μg mL^−1^), and the maximum absorption wavelength was determined. Gel cylinders were then incubated at 37 °C under constant shaking in 10 mL of pH 7.4 PBS or pH 5.5 acetate buffer, respectively. At predetermined intervals up to 168 h, 3 mL of medium was withdrawn and replaced with an equal volume of fresh buffer. The collected 3 mL sample was mixed with 3 mL of anhydrous ethanol to form a 50% ethanol mixture system, ensuring the released curcumin was completely dissolved. The absorbance of the mixture was measured by UV–Vis spectrophotometry, and the cumulative drug release percentage was calculated using the calibration curve prepared in the same 50% ethanol–buffer solvent. The drug content was quantified according to the standard curve regression equation. The cumulative release percentage at each time point was calculated using Equation (3).

(3) $$DR=\frac{M}{M_{0}},$$

where M is the cumulative amount released at time t, and M_0_ is the total drug mass loaded in the hydrogel.

#### 4.5.12. Cytotoxicity Study In Vitro

The in vitro cytotoxicity of hydrogel on BEAS-2B cells was evaluated using the CCK-8 assay kit. The cell viability was calculated using Equation (4).

(4) $$Cell\;Viability=\frac{A_{1}-A_{2}}{A_{3}-A_{2}},$$

where A_1_ is the absorbance of the test group treated with hydrogel extract, reflecting the material’s effect on cell activity; A_2_ is the absorbance of the blank group containing only CCK-8 reagent and culture medium, used for background correction; and A_3_ is the absorbance of the control group with cells cultured in normal medium, serving as the 100% viability baseline.

#### 4.5.13. Antibacterial Properties

The antibacterial performance of the CCM@OC hydrogel was comprehensively evaluated using a multi-dimensional approach. Against the Gram-negative model bacterium *Escherichia coli (E. coli*) and the Gram-positive model bacterium *Staphylococcus aureus* (*S. aureus*), three in vitro methods—optical density (OD) inhibition assay, plate colony counting, and scanning electron microscopy (SEM) morphology observation—were employed to assess antimicrobial activity.

After co-incubation of bacteria with hydrogels, the bacterial samples were harvested by centrifugation at 5000 rpm for 5 min, washed three times with PBS, and fixed with 2.5% glutaraldehyde at 4 °C overnight. Then, the fixed bacteria were dehydrated using a graded ethanol series (30%, 50%, 70%, 80%, 90%, 95%, and 100%) for 15 min each step, followed by vacuum drying. Finally, the samples were sputter-coated with gold and observed by SEM.

## 5. Conclusions

The substrate material UiO-66-NH_2_ was prepared by the solvothermal method. Cu-UiO-66-NH_2_ and Cur/Cu-MOF composites with both drug carrier function and metal synergism were obtained by a post-synthesis modification technique. Aldehyde modification of hyaluronic acid was carried out by sodium periodate oxidation. The OHA was further composited with carboxymethyl chitosan (CMCS) at a volume ratio of 1:2 to form a three-dimensional network structure by dynamic Schiff base bond cross-linking, and the composite hydrogel system was constructed by uniformly loading Cur/Cu-MOF; SEM observation showed that the gels were in a porous and interpenetrating network structure. The comprehensive performance evaluation of the Cur/Cu-MOF@OHA-CMCS hydrogel showed that the hydrogel exhibited excellent self-healing and injectability; it had good rheological and mechanical properties, and the equilibrium swelling rate reached 179% in the in vitro swelling experiment, which could effectively manage the wound exudate. The degradation behavior was pH-dependent, and the degradation rate was more than 60% for 12 h in the less-acidic environment; at the same time, the loading and release behavior of Cur showed excellent pH responsiveness, and the cumulative release amount could reach 71% for 7 d under the less-acidic condition (pH 5.5). Antibacterial experiments showed significant antibacterial performance against *E. coli* and *S. aureus*, and the CCK-8 test confirmed that the cell survival rate was >70%, confirming its highly efficient antibacterial properties and biosafety. In this work, a novel composite hydrogel system was constructed by innovatively combining a metal–organic framework with a dynamically cross-linked hydrogel, which achieved the organic integration of physical barrier construction, exudate management, smart drug release and antibacterial function through multi-component synergy and provided a new idea for the development of a new generation of smart wound dressings.

## Figures and Tables

**Table 1:** Gelation time with different volume ratios of OHA-CMCS hydrogels.

OHA:CMCS (*v*/*v*)	Gelation Time (s)
1:1	284 ± 15
1:2	238 ± 7
2:1	305 ± 4

## Data Availability

Data sharing is not applicable to this article as no datasets were generated or analyzed during the current study.

## Abbreviations

The following abbreviations are used in this manuscript:

Cur	Curcumin
HA	Hyaluronic Acid
OHA	Oxidized Hyaluronic Acid
CMCS	Carboxymethyl chitosan
MOF	Metal–Organic Framework
OD	Optical Density
PBS	Phosphate-Buffered Saline
DMF	*N*, *N*-dimethylformamide
UV	Ultraviolet
*E. coli*	*Escherichia coli*
*S. aureus*	*Staphylococcus aureus*
